# High efficiency photomodulators for millimeter wave and THz radiation

**DOI:** 10.1038/s41598-019-54011-6

**Published:** 2019-12-04

**Authors:** I. R. Hooper, N. E. Grant, L. E. Barr, S. M. Hornett, J. D. Murphy, E. Hendry

**Affiliations:** 1 Department of Physics and Astronomy, University of Exeter, Stocker Road, Exeter, Devon EX4 4QL United Kingdom; 2 School of Engineering, University of Warwick, Coventry, CV4 7AL United Kingdom

**Keywords:** Silicon photonics, Terahertz optics, Microwave photonics

## Abstract

Photomodulators for mm-wave and THz radiation are an essential component for many imaging and signal processing applications. While a myriad of schemes have been devised to enhance photomodulation by enhancing the light-matter interaction, there has been less focus on the photoconductive materials themselves, which are often the limiting factor. Here, we present an approach to increase the photomodulation efficiency of silicon by orders of magnitude, using post treatment of off-the-shelf silicon wafers. The increase in efficiency removes the need for bulky and costly amplified laser sources, and creates the potential for compact and cost-effective modulators for real-world applications. By passivating the surfaces of long bulk-lifetime silicon wafers with Al_2_O_3_, the recombination of the photoexcited carriers at the surfaces is mostly eliminated. This results in vastly longer excess carrier lifetimes (up to ~50 ms), with corresponding increases in photoconductivity. The resulting modulators are highly efficient, with the transmission through them being reduced from ~90% to <10% over a narrow frequency band with a continuous wave excitation intensity of just 10 Wm^−2^, whilst modulation factors of greater than 80% can be achieved over a broad band with similar intensities. We also discuss the limitations of such long-lifetime modulators for applications where the switching speed or spatial resolution of a modulator may be critical.

## Introduction

Despite many potential applications, the mm-wave and THz frequency bands have long been recognised as underutilised. Aiming to fill this “gap” in the spectrum, sources and detectors have become more readily available in recent years. However, devices to manipulate the radiation are still rather scarce and generally inefficient. For lower frequencies, electrical circuits can be used^1^, but these become progressively more expensive and lossy as the frequency increases, and are generally limited to frequencies <100 GHz. An overview of modulation techniques for frequencies >100 GHz, including all-optical, electronic and thermal-based modulators, can be found in the review article by Rahm *et al*.^2^. For these higher frequencies, all-optical modulators, whereby the mm-waves/THz are modulated by a secondary optical light source, are particularly attractive due to their relative simplicity. The potentially fast switching speeds of semiconductor-based photomodulators make them excellent candidates for signal processing in communication networks, as well as fast imaging^3–6^. Moreover, it is relatively straightforward to pattern the optical modulation and spatially control the scattered mm-waves/THz radiation^7^, and transient and tunable diffraction gratings and beam-steerers have been developed using this approach^8–11^, including using long carrier lifetime passivated silicon wafers^10,11^. Such modulators can be used to modulate signals across a broad spectrum from very low frequencies through to a few THz^6^.

Unfortunately, the photoconductivity of standard materials tends to saturate, limiting the efficiency of most photomodulators. Moreover, photomodulators fabricated from low photoconductivity materials require intense and expensive light sources, such as amplified femtosecond systems. While a myriad of schemes have been devised to boost photomodulation by enhancing the light matter interaction using unusual geometries e.g. prisms^12^, plasmon resonances^3^, metasurfaces^13–15^, and planar cavities^16^, there has been less focus on the semiconducting photoconductive materials themselves, which are often the limiting factor. In such materials, incident optical photons with energies greater than the bandgap generate free charge carriers. This increases the material’s conductivity and increases opacity to mm-wave/THz radiation. Silicon, as an indirect bandgap (long lifetime) semiconductor with relatively high mobility, is particularly appealing here, especially given its wide availability and relative low cost, though modulators based on other materials, such as germanium, have also been reported^17^. However, as we discuss further below, there is a huge range of modulation efficiencies reported in the literature for silicon^18–20^.

**Figure 1:**
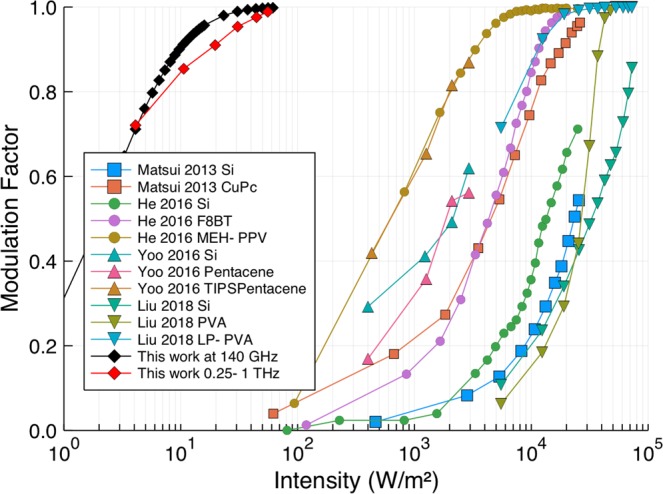
The modulation factor as a function of photoexcitation intensity as extracted from a number of recent papers in which the authors describe increased modulator efficiencies for coated silicon wafers (for details see the main text). Also shown are results from this work showing vastly improved efficiency from our passivated 675 μm thick wafer.

The efficiency of a modulator can be characterised by its transmission modulation factor,

1 $${\bf{MF}}=\frac{\Delta T(\omega )}{T(\omega )}$$

where *T* is the intensity transmitted through the unilluminated wafer and Δ*T* is the change in the transmitted intensity upon photoexcitation. For an “off-the-shelf” high resistivity silicon wafer, the conductivity change for a given illumination is rather small^18,19,21^. This necessitates large optical intensities to achieve significant modulation, typically ≫kW/m^2^. Achieving such high optical intensities requires high-power laser sources, which limits the application of mm-wave/THz systems outside of research laboratories. As such, in recent years, there have been increasing efforts to boost the efficiencies of silicon modulators through the application of various coatings, which have been shown to have surprisingly large effects. Figure 1 shows literature values for the modulation factor as a function of continuous wave (CW) optical pump intensity extracted from refs. ^18–22^, all of which use organic overlayers in an effort to increase the modulation factor. As well as organic overlayers, some success has been achieved by coating wafers with graphene, with Sensale-Rodriguez *et al*.^23^ reporting a 2.5 times improvement in modulator efficiency. A number of mechanisms for the observed increases in modulation factor have been suggested, but there is no clear consensus, and attempts to determine the underlying physics is further complicated by the wafer thickness, pump wavelength, and wafer growth type all varying between studies.

In this paper, by applying knowledge of charge carrier dynamics garnered by the solar cell community over many years, we show that the dominant effect is a reduction in the recombination rate of electron-hole pairs at the surface. By coating high bulk lifetime, high resistivity silicon wafers with state-of-the-art surface passivating layers, we demonstrate that this surface recombination can be effectively switched off. The resulting modulators are many orders of magnitude more efficient than those previously reported, displaying near complete switching for optical intensities of just 10 Wm^−2^. Finally, we discuss the limitations of such long-lifetime modulators in applications where the switching speed or spatial resolution is critical.

## Carrier Lifetime, Recombination, and Photomodulator Efficiency

### Photomodulator efficiency

When photons with energies above the bandgap of the silicon (1.1 eV) are absorbed they generate electron-hole pairs that will, on average, exist for a characteristic effective lifetime, *τ*_eff_, before recombining. In general, the steady-state excess carrier density will vary spatially (typically only a single surface of the wafer is illuminated), and the carrier distribution needs to be calculated using the full transport equations. However, if one is only interested in the transmission through the wafer, describing the system as a homogeneous layer with a uniform excess carrier density is generally a good approximation (this has been confirmed in comparisons with more detailed models - see Supplementary Information). In the steady-state this excess carrier density, Δ*n*, is given by,

2 $$\Delta n=G{\tau }_{{\rm{eff}}}$$

where *τ*_eff_ is the average lifetime of a charge carrier excited within the wafer, and *G* = *TI*/(*hfd*) is the generation rate of the electron-hole pairs (this is simply the areal number of charge carriers generated throughout the wafer divided by its thickness), and this assumes that the thickness of the wafer is greater than the penetration depth of the exciting light into the wafer. *G* itself is determined by the intensity of the incident radiation, *I*, the proportion of the incident photons that enter the wafer (given by the transmission, *T*, and calculated using the standard Fresnel equation for transmission through an interface), the thickness of the wafer, *d*, and the energy of the incident photons of frequency *f*. Thus, for a given illumination intensity, the only parameter of importance in determining the excess carrier concentration is the lifetime of the carriers; a longer lifetime results in a higher steady-state carrier density, which in turn results in a higher conductivity.

Note that the effective carrier lifetime is expected to be a function of carrier density, as will be described in more detail later, and that the constant effective lifetime used in this section is an approximation. However, the effective lifetime is relatively carrier density independent up to carrier densities of the order 1 × 10^21^ m^−3^, which is typically sufficient to achieve a modulation factor approaching 100%. For thin wafers (<200 μm) with long lifetimes (>10 ms) this approximation becomes increasingly less valid and the carrier density dependence of the effective lifetime would need to be included. Equation 2 would also no longer be valid.

We can model the influence of the lifetime on the modulation factor by incorporating the carrier density into a Drude model for both the electrons and holes to determine the dielectric permittivity, and subsequently use the Fresnel equation for a thin layer to calculate the transmission. The Drude model takes the form,

3 $${\varepsilon }_{{\rm{si}}}(\omega )={\varepsilon }_{{\rm{bg}}}-\frac{{\omega }_{pe}^{2}}{\omega (\omega +i{\gamma }_{e})}-\frac{{\omega }_{ph}^{2}}{\omega (\omega +i{\gamma }_{h})}$$

where *ω* is the angular frequency of the radiation, ${\varepsilon }_{{\rm{bg}}}=11.7+0.003i$ is the background permittivity of the silicon due to the lattice, ${\omega }_{p(e,h)}=\sqrt{({N}_{0(e,h)}+\Delta n){e}^{2}/({\varepsilon }_{0}{m}_{(e,h)})}$ are the plasma frequencies for the electrons and holes, with *m*_*e*_ = 0.26*m*_0_ and *m*_*h*_ = 0.38*m*_0_ being the conductivity effective masses (taken from^7^). *N*_0(*e*,*h*)_ are the thermal equilibrium electron and hole carrier densities (the non-photoexcited carrier densities), and will depend upon the doping level of the wafer. *γ*_(*e*,*h*)_ = *e*/(*m*_(*e*,*h*)_*μ*_(*e*,*h*)_), are the scattering rates of the electrons and holes, with *μ*_*e*_ = 0.145 m^2^ V^−1^s^−1^ and *μ*_*h*_ = 0.045 m^2^ V^−1^s^−1^ being the electron and hole mobilities (taken from^24^). *e* and ${\varepsilon }_{0}$ are the electron charge and permittivity of free space respectively. Note that the mobilities are also carrier density dependent at higher carrier densities^24,25^, but we omit this here as the variation is small over the range of densities achieved in this work (<3 × 10^21^ m^−3^). We note that, at the frequencies studied in this work, the change in the permittivity due to an increase in the carrier density is predominantly in its imaginary part. This is a result of the scattering frequency of both the electrons and holes being an order of magnitude greater than the frequency.

We can subsequently calculate the transmission through a wafer using the thin layer Fresnel equations,

4 $$t=\frac{{t}_{01}{t}_{12}\,\exp \,(i{k}_{z,si}d)}{1+{r}_{01}{r}_{12}\exp (i2{k}_{z,si}d)}$$

5 $$T=t{t}^{\ast }$$

where *t*_*ij*_ and *r*_*ij*_ are the single interface transmission and reflection Fresnel amplitude coefficients between the *i*^*th*^ and *j*^*th*^ layers, and *k*_*z*,*si*_ is the component of the wavevector of the incident light normal to the interface within the silicon. Here we will consider normal incidence and a free standing wafer in air so that ${t}_{01}=2/(1+\sqrt{{\varepsilon }_{si}})$, ${t}_{12}=2\sqrt{{\varepsilon }_{si}}/(1+\sqrt{{\varepsilon }_{si}})$, ${r}_{01}=-\,{r}_{12}=(1-\sqrt{{\varepsilon }_{si}})/(1+\sqrt{{\varepsilon }_{si}})$, and ${k}_{z,si}=\sqrt{{\varepsilon }_{si}}\omega /c$, where *c* is the speed of light in vacuum.

**Figure 2:**
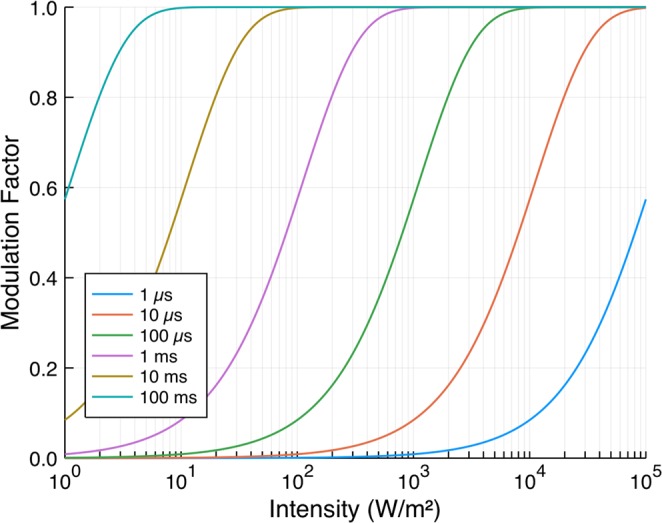
The modulation factor as a function of photoexcitation intensity for a range of effective carrier lifetimes as calculated from Eqs. 2–5 for 100 GHz radiation and a 500 μm thick wafer. The dominant factor in producing an efficient photomodulator is ensuring a high effective carrier lifetime.

The modulation factors for a range of effective carrier lifetimes as a function of the optical illumination intensity for 100 GHz radiation transmitted through a 500 μm thick undoped silicon wafer are shown in Fig. 2. The wavelength of the optical excitation used in the calculations was 625 nm, with a corresponding optical refractive index of the silicon of *n* = 3.89 + 0.017*i*. Undoped unpassivated “off-the-shelf” silicon wafers of typical thickness will have an effective carrier lifetime of the order of 10 μs, and the result shown here correlates well with the measured modulation factors from bare silicon wafers shown in Fig. 1.

It is clear that if one wishes to create more efficient modulators, one needs wafers with longer effective carrier lifetimes. To understand how one might go about achieving this, an understanding is required of the mechanisms by which the carriers recombine.

### Recombination processes in silicon

A detailed description of the carrier recombination processes in semiconductors can be found in many good semiconductor textbooks. In general, recombination can be expressed in terms of four independent processes: intrinsic Auger recombination, intrinsic band-to-band radiative recombination, defect-mediated Shockley-Read-Hall (SRH) recombination, and surface recombination^26^. Auger recombination is a three-carrier process which dominates at high carrier densities (typically greater than 10^21^ m^−3^). Band-to-band-radiative recombination is weak due to silicon’s indirect bandgap. SRH recombination, which can limit the lifetime at low carrier densities, occurs via defects (including impurities) in the bulk of the wafer, and hence is dependent on the quality of the wafer used. Surface recombination occurs due to the existence of defects, such as dangling bonds, at the sample surfaces. In an unpassivated high-purity silicon wafer the bulk lifetime can be many 10s of ms, whereas the surface lifetime is of the order of μs and dominates the effective lifetime (see Supplementary Information). These four contributions combine to give the effective lifetime,

6 $$\frac{1}{{\tau }_{{\rm{eff}}}}=\frac{1}{{\tau }_{{\rm{Auger}}}}+\frac{1}{{\tau }_{{\rm{radiative}}}}+\frac{1}{{\tau }_{{\rm{SRH}}}}+\frac{1}{{\tau }_{{\rm{surface}}}}$$

Thus, if one wishes to increase the effective lifetime of photoexcited carriers one must optimise the two non-intrinsic recombination processes. This means: (1) the quality of the wafer must be high to ensure the SRH recombination rate is as low as possible; and (2) the surface recombination rate must be minimised. The latter of these can be achieved through surface passivation.

### Surface passivation

The recombination rate at the surface is a product of the free carrier density at the surface, the areal density of interface states with energies within the bandgap, and the probability per unit time that an electron or hole will become trapped by a given level (dependent on the capture cross-section and the thermal velocity of that carrier). The latter two terms, when integrated over the bandgap energies, result in what is known as the fundamental surface recombination velocity, *S*, which encapsulates the effect of the surface on the recombination dynamics^27^. More generally, an effective surface recombination velocity, *S*_eff_, is defined which incorporates the excess carrier density at the surface and is the standard measure for describing the surface recombination rate.

Since the recombination rate at the surface depends upon both the carrier density at the surface and the density of traps at the surface, there are two main avenues for reducing it. Firstly, a dielectric layer can be added to the surface to terminate dangling bonds that arise due to the lattice termination. This gives a corresponding reduction in trap density and is known as chemical passivation. Historically silicon dioxide (SiO_2_) has been the most frequently used passivation layer, though there are other options^28–30^. The SiO_2_ is thermally grown on the Si surface, and terminates many of the dangling bonds. A subsequent annealing step in a gas mixture that contains hydrogen (e.g. forming gas) allows the hydrogen to diffuse through the SiO_2_ layer and terminate more of the dangling bonds. Reductions in surface trap densities of up to 5 orders of magnitude can be achieved in this way, reducing *S*_eff_ to sub-cm s^−1^ values and increasing the surface lifetime to tens of ms. This is comparable to the bulk SRH contribution in very good quality wafers, giving effective lifetimes of tens of ms.

The second passivation mechanism involves reducing the density of one of the carriers at the surface, and is known as “field effect passivation”^27^. Since both holes and electrons need to be present for recombination to occur, a region at the surface where there is a reduction in the concentration of one of the carriers will reduce recombination. This can be achieved by coating the wafer with a material with a fixed charge, and in recent years the use of aluminium oxide (Al_2_O_3_) grown by atomic layer deposition (ALD)^31,32^ has become popular due to its large fixed negative charge of approximately 2 × 10^16^ e m^−2^, where *e* is the elementary charge. This induces a large accumulation of holes near the surface, with a corresponding decrease in electron density, such that there are approaching 6 orders of magnitude fewer electrons than holes. The *S*_eff_ for these Al_2_O_3_ coated wafers can be as low 0.1 cm s^−1^, with effective lifetimes of over 50 ms achieved^33^. As a result, at lower carrier densities, the SRH recombination in the bulk of the wafer dominates and the effective lifetime is almost purely determined by the quality of the wafer.

## Surface Passivation and Characterisation

To demonstrate the expected increase in efficiency of silicon photomodulators upon surface passivation, we sourced commercially available, high-quality, high-resistivity, float-zone silicon wafers. We then passivated the surfaces with Al_2_O_3_, grown by atomic layer deposition, and tested their modulation efficiencies at mm-wave and THz frequencies. Three different wafers were tested: a 675 μm thick, 3 kΩ unpassivated wafer for comparison purposes; a second 675 μm thick wafer from the same batch that was subsequently passivated; and a 100 μm thick, 1 kΩ wafer that was also passivated. The 100 μm thick wafer was 100 mm in diameter and the 675 μm thick wafers were 150 mm in diameter. However, the surface passivated 675 μm thick wafer was cleaved to approximately 100 × 110 mm in order to fit the furnace used to activate the passivation.

Samples were subjected to a thorough surface preparation procedure developed previously^34^ which is critical to achieve excellent surface passivation. This involved a dip in HF (2%), an RCA 1 clean (H_2_O, H_2_O_2_ (30%), NH_4_OH (30%) in the ratio 5:1:1) at 75 °C for 5 minutes, a dip in HF (2%), a tetramethylammonium hydroxide etch at 80 °C for 10 minutes (removing 5 μm of silicon per side), a dip in HF (2%), an RCA 2 clean (H_2_O, H_2_O_2_ (30%), HCl (37%) in the ratio 5:1:1) at 75 °C for 10 minutes, and a final HF dip (2%). The samples were then pulled dry from the final HF dip (i.e. no rinsing) and were immediately transferred to a Veeco Fiji G2 ALD system. Al_2_O_3_ was deposited at 200 °C using a plasma O_2_ source and a trimethylaluminum precursor for 160 cycles to give films 15 nm thick on both sides. A post-deposition activation anneal was performed in a quartz tube furnace in air at 440 °C for 30 minutes.

**Figure 3:**
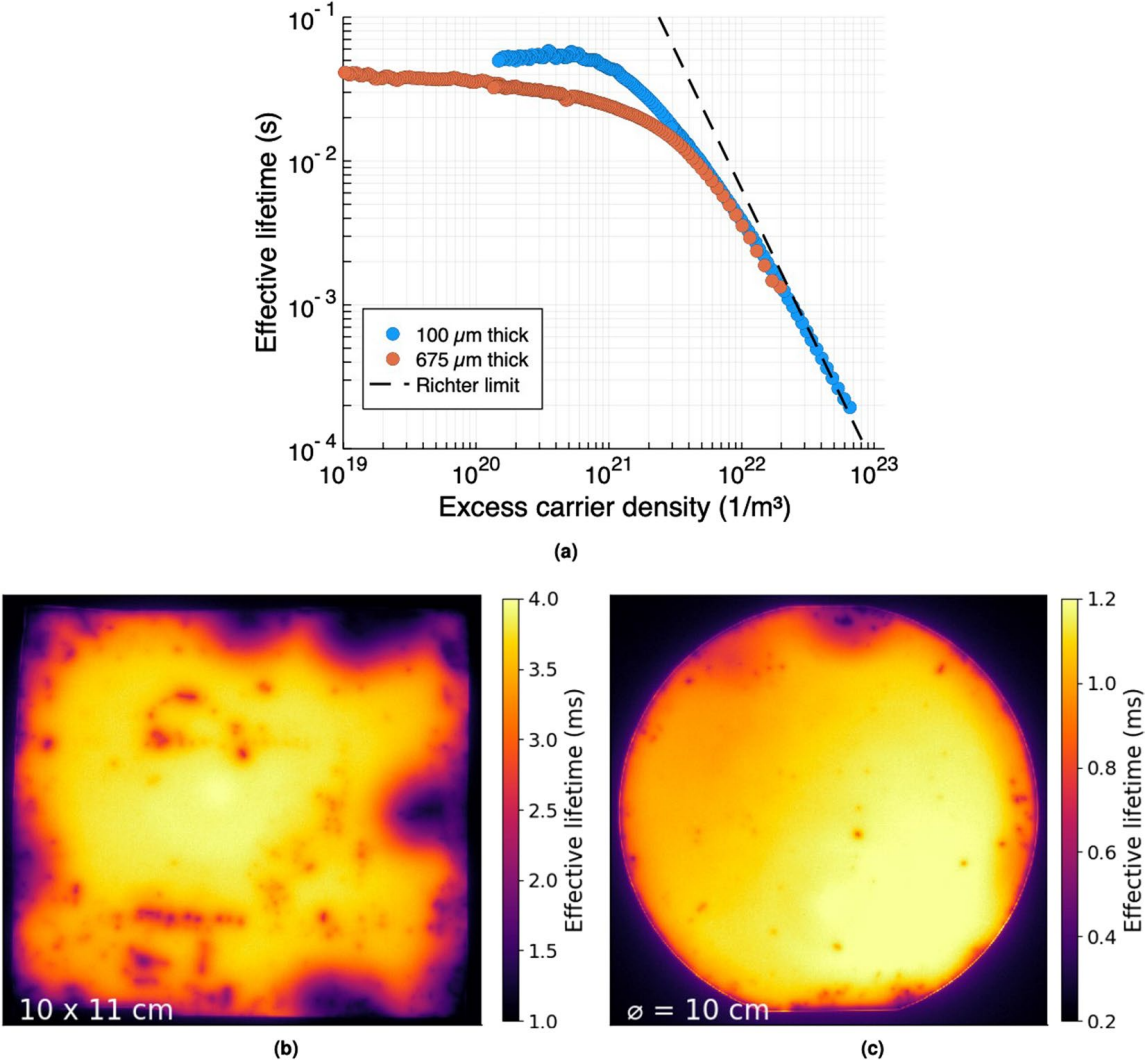
(**a**) The effective carrier lifetime as a function of the excess carrier density for a 3 kΩ 675 μm thick silicon wafer, and a 1 kΩ 100 μm thick silicon wafer. Both wafers were passivated with 15 nm of Al_2_O_3_. At low excess carrier densities the effective lifetime is relatively constant, with bulk SRH recombination dominating as the surfaces are well passivated. At higher densities intrinsic Auger recombination begins to dominate and the lifetime reduces. Also shown is the intrinsic lifetime limit of Richter *et al*.^37^, which is exceeded at high carrier densities as discussed in the text. (**b**,**c**) Effective carrier lifetime maps derived from photoluminescence images of of the passivated silicon wafers. The photoexcitation intensity was 1000 Wm^−2^ and the exposure time was 0.05 s. Image (**b**) is for the 675 μm thick wafer, and image (**c**) is for the 100 μm thick wafer.

After passivation, the effective lifetime of the wafers was measured using transient photoconductance decay^35^ using a Sinton WCT-120 lifetime tester, with results shown in Fig. 3a. The uniformity of the passivation was determined via photoluminescence (PL) imaging^36^ using a BT Imaging LIS-L1 system and light emitting diode (LED) array to excite the sample. The results, using an illumination intensity of 1,000 Wm^−2^ and an exposure time of 0.05 s, are shown in Fig. 3b,c. Excellent uniformity of carrier lifetime is seen in the 100 μm thick wafer. The 675 μm thick wafer is less uniform, particularly around the edges, as a result of damage to the Al_2_O_3_ passivation layer during handling.

The lifetime curves shown in Fig. 3a represent the current state-of-the-art for ALD Al_2_O_3_ passivation^33^. An upper limit for the surface recombination velocity with our passivation scheme can be obtained by assuming an infinite bulk lifetime. At an excess carrier density of 10^21^ m^−3^, the effective lifetime in the 100 μm thick wafer is 44 ms, which corresponds to a worst case *S*_eff_ of 0.11 cm s^−1^. For low carrier densities, the lifetime is relatively independent of carrier density and is determined by low levels of bulk SRH recombination, whereas at higher carrier densities Auger recombination begins to dominate and the lifetime reduces rapidly. Both wafers exhibit effective lifetimes of the order of 30–50 ms at low carrier densities, thus making them excellent candidates for efficient photomodulators. The thicker wafer has a lower effective lifetime than the thinner one, and we attribute this to higher levels of bulk SRH recombination in the former. It is noted that at very high excess carrier densities the experimentally measured effective lifetime exceeds the so-called intrinsic limit of Richter *et al*.^37^. This again is testament to the quality of the surface passivation, and highlights the conservative nature of the currently accepted intrinsic limit, as has recently become widely acknowledged elsewhere^33,38,39^.

## Photomodulation

**Figure 4:**
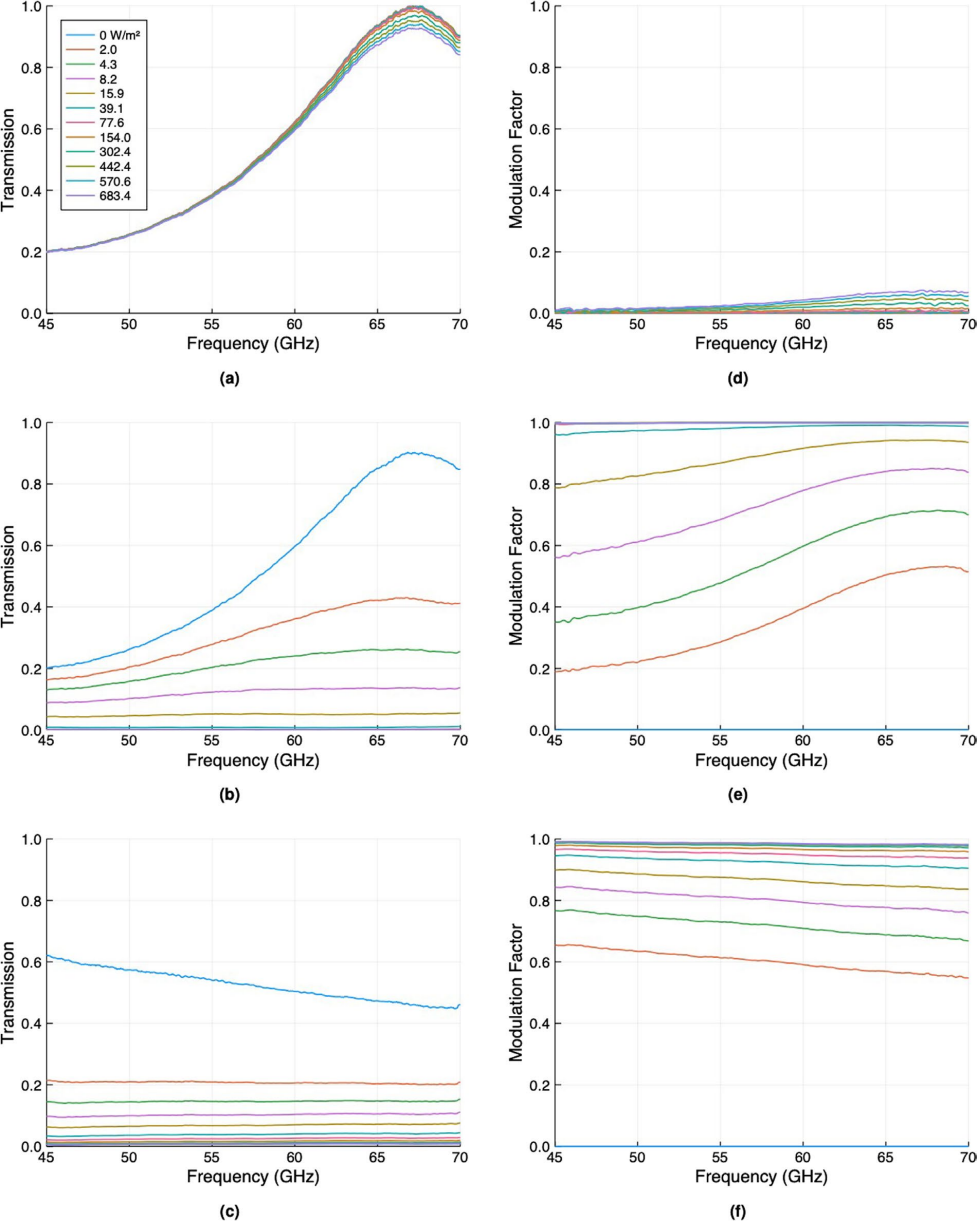
(**a**–**c**) The s-polarised transmission as a function of frequency for various photoexcitation intensities through an unpassivated 675 μm thick silicon wafer, a passivated 675 μm thick wafer from the same batch, and a passivated 100 μm thick wafer. The wafers were oriented at 45 degrees to both the longer-wavelength radiation and the photoexciting light. (**d**–**f**) The corresponding modulation factors as calculated from (**a**–**c**).

**Figure 5:**
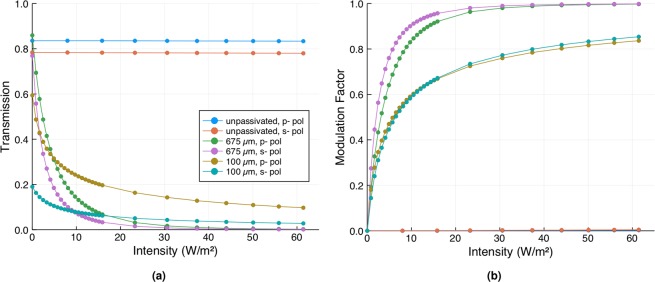
(**a**) The transmission at a 45 degree angle of incidence of 140 GHz radiation through the unpassivated 675 μm thick wafer, and the passivated 100 μm and 675 μm thick wafers, as a function of photoexcitation intensity and for both s- and p-polarisations. (**b**) The corresponding modulation factors as calculated from (**a**).

The modulation factor at mm-wave frequencies was measured using two different experimental set-ups. An Anritsu Vectorstar MS4647B Vector Network Analyser was used to measure the transmission through the wafers for s-polarised light over a frequency range of 45–70 GHz. The radiation was emitted and detected using Flann 25240 25 dB standard gain horn antennas. The wafers were photoexcited using a collimated 4.8 W SOLIS-623C LED from Thorlabs with an output wavelength of 623 nm. The mm-wave and optical beams were at right angles to each other, with the wafers oriented at 45 degrees to both and placed at their intersection such that both pump and probe beams overlapped. The transmission as a function of frequency was measured for various excitation intensities and converted into a modulation factor. These are shown in Fig. 4. The modulation factor was also measured at 140 GHz, using an IMPATT diode source and detector from Terasense, in a similar geometry as the mm-wave measurements, using two PTFE lenses (10 cm) to refocus the divergent output of the IMPATT diode to a 2.5 cm spot on the sample and to collect the transmitted radiation. The 140 GHz transmission through the wafer and the corresponding modulation factors for a range of excitation intensities and for both p- and s-polarisations are shown in Fig. 5.

For the unpassivated 675 μm thick wafer (Fig. 4a) there is a clear resonance in the transmission at approximately 67 GHz due to a Fabry-Pérot condition related to the wafer thickness. When illuminated with the optical light there is a decrease in the transmission as free charge carriers are generated, as expected. When this transmission is converted to a modulation factor we note that the results are comparable to those of bare silicon wafers tested by Matsui and He (see Fig. 1).

For the passivated 675 μm thick wafer (Fig. 4b) the results are dramatically different. Firstly, we note a small reduction in the peak height of the Fabry-Pérot resonance, even without optical illumination. This is a result of the thin (<100 nm) layer of increased charge density at the surface induced by the field-effect passivation (note that our simple model of a uniform carrier density used to generate the plots in Fig. 2 is therefore not totally accurate here - see section 3 of the Supplementary Information for more details). Nevertheless, we see large changes upon photoexcitation - at the Fabry-Pérot resonance an illumination intensity of just 2 Wm^−2^ is sufficient to halve the transmitted intensity through the wafer. This is an extremely low light intensity, approximately 1/500^th^ of strong daylight at zenith. A further increase in the optical intensity to just 40 Wm^−2^ is sufficient to saturate the modulation, with a total change in transmission on resonance through the wafer of over 90%. By comparison (from Fig. 2), to achieve the same modulation factor in an unpassivated wafer with an effective lifetime of the order of 10 μs, an optical intensity approaching 40 kWm^−2^ would be required.

To improve modulator efficiency over a broad frequency band one needs to employ a different strategy. Here, a very sub-wavelength wafer thickness is required, as is shown in Fig. 4c. For this passivated 100 μm thick wafer, transmission is high for low frequencies. Moreover, higher carrier densities can be achieved in a thinner wafer for a given photogeneration rate. These two effects combine to give a large modulation factor over a broad frequency range, with improved performance as the frequency is reduced. For comparison, at the lowest frequency studied here (45 GHz) the modulation factor for our lowest excitation intensity of 2 Wm^−2^ is 0.65 for the passivated 100 μm thick wafer as compared to 0.2 for the 675 μm thick wafer. More importantly, there is an increase in the change in transmission to 0.4 from just 0.04.

At 140 GHz, see Fig. 5, similar results are evident. The passivated 675 μm thick wafer displays the more efficient modulation: at the 45 degree angle of incidence, 140 GHz lies close to the 2^nd^ order Fabry-Pérot resonance of this wafer. However, since it is not exactly at the resonant condition the efficiency of photomodulation is reduced somewhat. There is also a clear difference between the two polarisations. This is most obvious in the transmission through the passivated 100 μm thick wafer, where the overall change in transmission for p-polarised light is almost three times that for s-polarised light, even though their modulation factors are very similar. Depending upon the application and the possible illumination geometry of the modulator, p-polarisation could be utilised to enhance performance for off-angle illumination.

**Figure 6:**
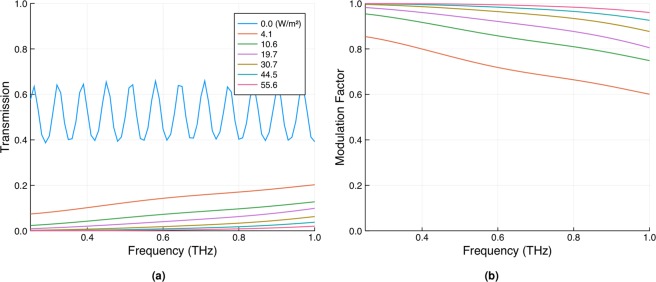
(**a**) The normal incidence THz transmission through a passivated 675 μm thick silicon wafer for various photoexcitation intensities. (**b**) The corresponding modulation factors as calculated from (**a**). The average value of the transmission across the frequency range for the un-photoexcited data was used to calculate the modulation factors.

Whilst thus far we have characterised our modulators in the mm-wave band, it is important to note that efficient modulation can be achieved at frequencies up to and beyond 1 THz. To demonstrate this we used THz time-domain spectroscopy (specifically, the setup described in the supplementary information of ref. ^40^) to measure the normal incidence transmission through our passivated 675 μm thick wafer for various optical illumination intensities (see Fig. 6). A series of higher-order Fabry-Pérot resonances are clearly evident in the un-photoexcited data (though somewhat shallowed by the ≈20° angular dispersion of the THz beam). Note that these are absent in the photoexcited data, partly as a result of a lower frequency resolution in these measurements (required to minimise the effects of rotational water absorption, as physical constraints in the experiment necessitated measurement in an open environment), but mostly as a result of the increased absorption upon photoexcitation. However, the most important observation is that, whilst it is clear that modulator efficiency becomes progressively worse as the frequency increases (as expected from the frequency dependence of the Drude model), close to 100% modulation can still be achieved for illumination intensities of just 10s of Wm^−2^ up to and beyond 1 THz.

## Limitations

The results shown here demonstrate that passivating the surface of high bulk lifetime silicon wafers to reduce surface recombination vastly enhances their efficiency as photomodulators, with efficiencies approaching 4 orders of magnitude greater than those of off-the-shelf wafers. However, there are trade-offs. The increased lifetime in passivated wafers will also give a correspondingly longer switching time, and thus for some applications may not be appropriate. From Fig. 2, one can choose the required lifetime to achieve a certain switching speed, and subsequently determine the excitation intensity needed to achieve a given modulation factor. One can therefore, in principal, engineer the switching time and modulation factor by systematically controlling *τ*_eff_. However, this tunability in lifetime is an experimental challenge which is not yet addressed.

In addition, if one requires a spatially patterned conductivity profile, e.g. for imaging or tunable beam-steerers, there is a second trade-off. Long lifetimes result in longer carrier diffusion lengths, given by $L=\sqrt{D{\tau }_{{\rm{eff}}}}$, where *D* is once again the ambipolar diffusion constant. For lifetimes of approximately 30 ms, as in the wafers used in this study, the diffusion length is around 7 mm. This is likely too long relative to the wavelength for use in mm-wave/THz spatial modulators. Here, again, one can look to tunability in lifetime: Eqs. 2–5 can be used, along with Fig. 2, to determine the excitation intensity needed to give a particular modulation factor whilst maintaining the required spatial resolution.

Thus there is a fundamental compromise between long carrier lifetimes for efficient modulation, and short carrier lifetimes for fast switching and minimal carrier diffusion. The ability to select an arbitrary carrier lifetime, tuned to any particular application, would therefore be of great benefit. The most straightforward route to this is by varying the starting conditions: off-the-shelf silicon wafers often exhibit much faster bulk SRH recombination than the high quality wafers used in this study. Since, once fully passivated, SRH recombination sets the effective carrier lifetime, surface passivation of poorer quality wafers should in principal allow for such lifetime engineering. However, we note that the bulk lifetimes of off-the-shelf wafers are currently not usually specified, especially for poorer quality wafers.

## Conclusion

In this paper we have described silicon photomodulators that approach being four orders of magnitude more efficient than off-the-shelf, unpassivated wafers, and around two orders of magnitude more efficient than other silicon based photomodulators described in the literature. The critical property is the effective lifetime of the photoexcited charge carriers, and this is dominated by recombination processes at the surface. By passivating the surface of high bulk lifetime wafers with atomic layer deposited Al_2_O_3_, as used in state-of-the-art, high efficiency, solar cells, this effective lifetime can be increased from approximately 10 μs to several 10s of ms. This increase results in considerably more efficient modulation and could eliminate the need for intense laser sources in applications where fast switching speeds/high spatial resolution are not required. However, it is clear that the inherent trade-off between efficiency and switching speed/diffusion length will severely limit the utility of very high-efficiency modulators. By demonstrating the current efficiency limit of silicon-based photomodulators, whilst also discussing their inherent limitations, we hope that applied physicists and engineers will be able to make informed choices regarding their potential use in applications.

## Supplementary information

Supplementary Information
