# Supplementary material for: High efficiency photomodulators for millimeter wave and THz radiation
Source: Sci Rep. 2019 Dec 4;9:18304. doi: 10.1038/s41598-019-54011-6 (PMC6893041; doi:10.1038/s41598-019-54011-6)
Supplement: Supplementary file 1 — Supplementary Information [file 41598_2019_54011_MOESM1_ESM.pdf]

Supplementary information: High efficiency photomodulators for millimeter wave and THz radiation

I. R. Hooper[1,*], N. E. Grant[2], L. E. Barr[1], S. M. Hornett[1], J. D. Murphy[2], and E. Hendry[1]

[1]Department of Physics and Astronomy, University of Exeter, Stocker Road, Exeter, Devon, EX4 4QL, United Kingdom
[2]School of Engineering, University of Warwick, Coventry, CV4 7AL, United Kingdom
[*]i.r.hooper@exeter.ac.uk

1 Introduction

In this supplementary information we initially introduce a diffusion model for photoexcited charge carriers in silicon that will be used to obtain model results for subsequent sections. We also describe how the carrier distributions obtained from this model can be used to calculate the transmission of longer wavelength radiation through photoexcited wafers.

In section 3 we show that calculating the transmission of radiation through a silicon wafer by describing the dielectric function as spatially uniform within the wafer, and determined using the average charge carrier density, is a very good approximation, and that using far more complex charge carrier transport models to calculate the spatially varying carrier distribution is, in general, not necessary. This justifies our use of Eqn. 2 and the subsequent theory in the accompanying article.

Subsequently, in section 4, we use our diffusion model to show that recombination of photoexcited charge carriers at the surface dominates the effective carrier lifetime, justifying claims made in the text surrounding Eqn. 6 in the accompanying article.

2 The Model

Our starting point is the 1D diffusion equation describing the evolution of the distribution of excess charge carriers within the wafer,

$$\frac{\partial N(z,t)}{\partial t} = D\frac{\partial^2 N(z,t)}{\partial z^2} + G(z,t) - \frac{N(z,t)}{\tau_\mathrm{b}} \tag{1}$$

where $N(z,t)$ is the excess carrier density, D is the ambipolar diffusion coefficient (taken as $D = 0.0017$ m^2/s from [1]), $G(z,t)$ is the generation rate due to photoexcitation, and τ_b is the effective lifetime in the bulk of the wafer. We note that this model includes only the Shockley-Read-Hall contribution to the bulk lifetime, and ignores radiative and Auger recombination. Within the range of carrier densities encountered in this work the contribution due to radiative recombination can easily be discounted as having no significant impact. However, since Auger recombination begins to dominate at carrier densities greater than approximately $10^{21}\,m^{-3}$ (see Fig. 3 in accompanying article), any results presented later in this supplementary information showing carrier densities greater than this will be incorrect to some degree. The crux of the arguments, however, will not be altered.

In addition to recombination in the bulk, we also have recombination at the surface, which can be described by the following boundary condition at the front and back interfaces,

$$D\frac{\partial N(z)}{\partial z} = S_{0,1}N_{z=1,d} \tag{2}$$

where $S_{0,1}$ are the surface recombination velocities at the front and back faces of the wafer, respectively.

In the subsequent subsections we will describe the form of the generation rate, the method used to calculate the steady-state carrier profile, the Drude model used to extract the profile of the permittivity through the wafer, and the method used to subsequently determine the transmission of longer-wavelength radiation through the wafer.

2.1 The generation rate, G

When illuminated with light with photon energies greater than the band gap, photoexcited carriers are generated with a distribution determined by the penetration of the exciting light into the wafer. Here, and throughout this document, we will consider the simple case of a free standing silicon wafer (medium 2) bounded by air (media 1 and 3), and photoexcited with light at normal incidence.

The generated carriers are excited with a distribution given by,

$$G(z) = \frac{\kappa_a I T_{12} \lambda_{\rm exc}}{hc} \left(\frac{\exp(-\kappa_a z) + R_{23} \exp(\kappa_a z) \exp(-2\kappa_a d)}{1 - R_{21} R_{23} \exp(-2\kappa_a d)} \right) \tag{3}$$

where the multiplying term gives the number of electron-hole pairs generated at the illuminated interface and is given by the proportion of light transmitted into the wafer T_{12}, the absorption coefficient, $\kappa_a = 4\pi k/\lambda_{\rm exc}$, where $\lambda_{\rm exc}$ is the wavelength of the exciting light and k is the imaginary part of the refractive index at that wavelength, by the intensity of the illuminating light, I, and the energy of each photon, $hc/\lambda_{\rm exc}$. The term in the brackets describes the distribution within the wafer including multiple reflections from the front and back faces. For wafers much thicker than the penetration depth (typically the case) this term reduces to $\exp(-\kappa_a z)$. d is the thickness of the wafer, and R_{21} and R_{23} are the intensities of the light reflected from the front and back interfaces of the wafer when incident from medium 2 to media 1 and 3 respectively. Note that the above equation is only valid if the coherence length of the photoexciting light is much shorter than the thickness of the wafer.

The T_{ij} and R_{ij} terms are calculated using the standard Fresnel equations,

$$\begin{aligned} R_{ij} &= (1 - \sqrt{\varepsilon_{\rm si}})^2 / (1 + \sqrt{\varepsilon_{\rm si}})^2 \\ T_{ij} &= 4\sqrt{\varepsilon_{\rm si}} / (1 + \sqrt{\varepsilon_{\rm si}})^2 \end{aligned} \tag{4}$$

where $\varepsilon_{\rm si}$ is the dielectric constant of the silicon at the wavelength of the photoexciting light.

In order to calculate the spatial distribution of the carrier generation we must incorporate the multiple reflections of the photoexciting light within the wafer. For brevity we will call the multiplying term in Eqn. 3, A. Our series of multiple reflections is then given by,

$$\begin{aligned} G(z) = {} & A \exp(-\kappa_a z) \\ & + A \exp(-\kappa_a d) R_{23} \exp(-\kappa_a (d - z)) \\ & + A \exp(-\kappa_a d) R_{23} \exp(-\kappa_a d) R_{21} \exp(-\kappa_a z) \\ & + A \exp(-\kappa_a d) R_{23} \exp(-\kappa_a d) R_{21} \exp(-\kappa_a d) R_{23} \exp(-\kappa_a (d - z)) \\ & + A \exp(-\kappa_a d) R_{23} \exp(-\kappa_a d) R_{21} \exp(-\kappa_a d) R_{23} \exp(-\kappa_a d) R_{21} \exp(-\kappa_a z) \\ & + ... \end{aligned} \tag{5}$$

which, after some simple manipulations, reduces to,

$$\begin{aligned} G(z) = {} & A(\exp(-\kappa_a z) + R_{23} \exp(-2\kappa_a d) \exp(\kappa_a z)) \\ & \times [1 + R_{23} R_{21} \exp(-2\kappa_a d) + R_{23}^2 R_{21}^2 \exp(-4\kappa_a d) \\ & + R_{23}^3 R_{21}^3 \exp(-6\kappa_a d) + ...] \end{aligned} \tag{6}$$

The term in the square brackets is a geometric series $1 + z + z^2 + z^3 + ...$ with $z = R_{12} R_{01} \exp(-2\kappa_a d)$. Recalling that the sum of such a geometric series is $1/(1 - z)$, we get,

$$G(z) = A \left(\frac{\exp(-\kappa_a z) + R_{23} \exp(\kappa_a z) \exp(-2\kappa_a d)}{1 - R_{21} R_{23} \exp(-2\kappa_a d)} \right) \tag{7}$$

2.2 Implementing the Finite Difference Method - Steady State Solution

To obtain the steady-state charge carrier density profile when the wafer is being continuously photoexcited we simply set the time derivative in (1) to zero, giving,

$$0 = D\frac{\partial^2 N(z)}{\partial z^2} + G(z) - \frac{N(z)}{\tau_b} \tag{8}$$

and solve this system using the finite difference method. This involves 3 main steps: 1) Discretise the problem and describe the differentials in an appropriate way, 2) Convert this into a matrix system taking into account the appropriate boundary conditions, and 3) Solve the matrix system.

We discretise our 1D system into a uniform grid of points, and recall that, for a twice differentiable function, we have,

$$\phi''(z) \approx \frac{\phi(z-\Delta z) - 2\phi + \phi(z+\Delta z)}{(\Delta z)^2} \tag{9}$$

Thus we can rewrite our diffusion equation as,

$$D\frac{N_{i+1} + N_{i-1} - 2N_i}{\Delta z^2} - \frac{N_i}{\tau_b} = -G_i \tag{10}$$

which in matrix form becomes,

$$\begin{bmatrix} G_1 \\ G_2 \\ G_3 \\ \vdots \\ G_{n-1} \\ G_n \end{bmatrix} = \begin{bmatrix} \frac{2D}{(\Delta z)^2} + \frac{1}{\tau_b} & -\frac{D}{(\Delta z)^2} & 0 & 0 & 0 \\ -\frac{D}{(\Delta z)^2} & \frac{2D}{(\Delta z)^2} + \frac{1}{\tau_b} & -\frac{D}{(\Delta z)^2} & 0 & 0 \\ 0 & -\frac{D}{(\Delta z)^2} & \frac{2D}{(\Delta z)^2} + \frac{1}{\tau_b} & -\frac{D}{(\Delta z)^2} & 0 \\ \ddots & \ddots & \ddots & \ddots & \ddots \\ 0 & 0 & -\frac{D}{(\Delta z)^2} & \frac{2D}{(\Delta z)^2} + \frac{1}{\tau_b} & -\frac{D}{(\Delta z)^2} \\ 0 & 0 & 0 & -\frac{D}{(\Delta z)^2} & \frac{2D}{(\Delta z)^2} + \frac{1}{\tau_b} \end{bmatrix} \begin{bmatrix} N_1 \\ N_2 \\ N_3 \\ \vdots \\ N_{n-1} \\ N_n \end{bmatrix} \tag{11}$$

To implement our boundary conditions (Eqn.(2)) we use the ghost point method to ensure we maintain second order accuracy.

We create "ghost" points outside of our domain at $z = 0$ and $z = n + 1$. At $z = 1$ we use the central difference equation for the differential, giving,

$$D\left(\frac{N_2 - N_0}{2\Delta z}\right) = S_0 N_1 \tag{12}$$

and subsequently substitute this into the equivalent central FD equation (9), getting

$$-\left(\frac{2}{(\Delta z)^2} + \frac{S_0}{\Delta z} + \frac{1}{\tau_b}\right)N_1 + \frac{2N_2}{(\Delta z)^2} = -G_1 \tag{13}$$

with an equivalent equation for the 2nd boundary. Incorporating these into the matrix system results in,

$$\begin{bmatrix} G_1 \\ G_2 \\ G_3 \\ \vdots \\ G_{n-1} \\ G_n \end{bmatrix} = \begin{bmatrix} \frac{2D}{(\Delta z)^2} + \frac{2S_0}{\Delta z} + \frac{1}{\tau_b} & -\frac{2D}{(\Delta z)^2} & 0 & 0 & 0 \\ -\frac{D}{(\Delta z)^2} & \frac{2D}{(\Delta z)^2} + \frac{1}{\tau_b} & -\frac{D}{(\Delta z)^2} & 0 & 0 \\ 0 & -\frac{D}{(\Delta z)^2} & \frac{2D}{(\Delta z)^2} + \frac{1}{\tau_b} & -\frac{D}{(\Delta z)^2} & 0 \\ \ddots & \ddots & \ddots & \ddots & \ddots \\ 0 & 0 & -\frac{D}{(\Delta z)^2} & \frac{2D}{(\Delta z)^2} + \frac{1}{\tau_b} & -\frac{D}{(\Delta z)^2} \\ 0 & 0 & 0 & -\frac{2D}{(\Delta z)^2} & \frac{2D}{(\Delta z)^2} + \frac{2S_1}{\Delta z} + \frac{1}{\tau_b} \end{bmatrix} \begin{bmatrix} N_1 \\ N_2 \\ N_3 \\ \vdots \\ N_{n-1} \\ N_n \end{bmatrix} \tag{14}$$

which can be solved to obtain $N(z)$ in the usual way, and added to the intrinsic carrier density N_0 to obtain the spatial dependence of the total carrier density,

$$N_{tot}(z) = M^{-1}G(z) + N_0 \tag{15}$$

2.3 The Dielectric Function

Once the spatial dependence of the carrier distribution has been determined, it is a simple matter to obtain the spatially dependent permittivity profile through the wafer.

We note that we have both electrons and holes as free charges, and we will assume that we have equal quantities of each given by $N_{tot}(z)$. We can then calculate the dielectric permittivity using a Drude model,

$$\varepsilon(\omega, z) = \varepsilon_{\rm bg} - \frac{\omega_{pe}(z)^2}{\omega(\omega + i\gamma_e)} - \frac{\omega_{ph}(z)^2}{\omega(\omega + i\gamma_h)} \tag{16}$$

where ω is the angular frequency of the radiation, $\varepsilon_{\rm bg} = 11.7 + 0.003i$ is the background permittivity of the silicon due to the lattice (though this does depend somewhat upon the quality of the silicon, in particular in the imaginary part), $\omega_{p(e,h)} = \sqrt{N_{tot}e^2/\left(\varepsilon_0 m_{(e,h)}\right)}$ are the plasma frequencies for the electrons and holes, with $m_e = 0.26m_0$ and $m_h = 0.38m_0$ being the conductivity effective masses (taken from [2]). $\gamma_{(e,h)} = e/\left(m_{(e,h)}\mu_{(e,h)}\right)$ are the scattering rates of the electrons and holes, with $\mu_e = 0.145$ m^{2}V^{-1}s^{-1} and $\mu_h = 0.045$ m^{2}V^{-1}s^{-1} being the electron and hole mobilities (taken from [3]). e and ε_0 are the electron charge and permittivity of free space respectively.

Note that the mobilities are also carrier density dependent at higher carrier densities (see references in accompanying article), but we omit this here as the variation is small over the range of densities achieved in this work.

2.4 Transmission and Reflection Calculations

In order to calculate the reflection and transmission through the discretised system we use the transfer matrix method. The fields on either side of a layer can be related by a transfer matrix, M,

$$\begin{bmatrix} E_{j-1,j} \\ \mu_0 H_{j-1,j} \end{bmatrix} = \begin{bmatrix} \cos(\beta_j) & i\frac{\sin(\beta_j)}{\xi_j} \\ i\xi_j \sin(\beta_j) & \cos(\beta_j) \end{bmatrix} \begin{bmatrix} E_{j,j+1} \\ \mu_0 H_{j,j+1} \end{bmatrix} \tag{17}$$

where $\beta_j = k_{z,j}\Delta z$, $k_{z,j} = \sqrt{\varepsilon_j k_0^2 - k_x^2}$ is the normal component of the wavevector in layer j (k_x is the in-plane wavevector), and $\xi_j = k_{z,j}$ for s-polarised light and $\xi_j = k_{z,j}/\varepsilon_j$ for p-polarised light.

To calculate the transfer matrix for the whole system one simply multiplies the transfer matrices of the j layers,

$$\begin{bmatrix} E_0 \\ \mu_0 H_0 \end{bmatrix} = M_1 M_2 ... M_N \begin{bmatrix} E_{N+1} \\ \mu_0 H_{N+1} \end{bmatrix} = M_{tot} \begin{bmatrix} E_{N+1} \\ \mu_0 H_{N+1} \end{bmatrix} \tag{18}$$

where the subscripts 0 and $N+1$ for E and H correspond to the media on the incident and exit sides of the wafer respectively.

We can then identify the fields on either side of the stack with incident reflected and transmitted field amplitudes, giving,

$$\begin{bmatrix} E_0 \\ \mu_0 H_0 \end{bmatrix} = \begin{bmatrix} E_i + E_r \\ (E_i - E_r)\xi_0 \end{bmatrix} = M_{tot} \begin{bmatrix} E_t \\ \xi_{N+1} E_t \end{bmatrix} = M_{tot} \begin{bmatrix} E_{N+1} \\ \mu_0 H_{N+1} \end{bmatrix} \tag{19}$$

where E_i, E_r, and E_t are the field amplitudes of the incident, reflected, and transmitted waves respectively.

From this, one can determine the reflection and transmission amplitude coefficients from the elements of the total transfer matrix,

$$r = \frac{\xi_0 m_{11} + \xi_0 \xi_{N+1} m_{12} - m_{21} - \xi_{N+1} m_{22}}{\xi_0 m_{11} + \xi_0 \xi_{N+1} m_{12} + m_{21} + \xi_{N+1} m_{22}} \tag{20}$$

$$t = \frac{2\xi_0}{\xi_0 m_{11} + \xi_0 \xi_{N+1} m_{12} - m_{21} - \xi_{N+1} m_{22}} \tag{21}$$

Since we will only consider normal incidence in this document, the reflected and transmitted intensities are simply given by $R = rr^*$ and $T = tt^*$.

3 Using the average carrier density to calculate the transmission of radiation through a wafer

In the accompanying article we state that calculating the transmission of longer-wavelength radiation through a wafer by considering it as a slab of material with a uniform carrier density (given by the average carrier density) is a good approximation. In this section we demonstrate this by calculating the transmission through wafers using the above theory and comparing it to the results when we use only the average carrier density. In Fig. S1 we show the spatially dependent charge carrier densities through a silicon wafer for a range of surface recombination velocities, and for excitation wavelengths of 400 nm, 625 nm, and 1000 nm, and for wafer thicknesses of 100 µm and 675 µm. The background electron and hole carrier density, N_0, is low since we are using high resistivity silicon. Here we use $N_0 = 10^{15}$ m^{-3}, but any value below this makes no discernible difference to the transmission. The optical refractive indices for each wavelength were taken from [4], and take the values $n_{400nm} = 5.62 + 0.33i$, $n_{625nm} = 3.89 + 0.017i$, and $n_{1000nm} = 3.58 + 0.0005i$, and the bulk lifetime, τ_{b} is 10 ms in all cases. The photoexcitation intensity was 100 Wm^{-2}, and the wafers were illuminated from the left hand side. We also assume that the surface recombination velocity is the same on both interfaces.

Important aspects to note are that: 1. For all cases, at low S, the carrier density throughout the wafer is rather uniform, and as such we would expect our approximation of using the average carrier density in our transmission calculations to be valid. 2. At higher surface recombination velocities, however, there can be carrier density variations throughout the wafer of approaching 2 orders of magnitude. 3. There is an increase in carrier density at high surface recombination velocity when the wafers are photoexcited with light with energies closer to the band gap. This is due to the longer penetration depth into the silicon, resulting in a larger proportion of charge carriers being excited away from the low carrier lifetime region at the surface.

To test the validity of using the average carrier density when calculating the transmission through a wafer, we first calculate the transmission as per the theory above using the spatially varying carrier density profile. We then calculate the average carrier density from those profiles and use that to determine the average permittivity of the silicon using the Drude model in Eqn. 16. The transmission through the slab is then given by the standard Fresnel equation,

$$t = \frac{t_{12} t_{23} \exp(i k_{z,\mathrm{si}} d)}{1 + r_{12} r_{23} \exp(i 2 k_{z,\mathrm{si}} d)} \tag{22}$$

$$T = tt^* \tag{23}$$

where t_{ij} and r_{ij} are the single interface transmission and reflection Fresnel amplitude coefficients between the ith and j^{th} layers, and $k_{z,\mathrm{si}}$ is the component of the wavevector of the incident light normal to the interface within the silicon. Here we will consider normal incidence and a free standing wafer in air so that $t_{12} = 2\sqrt{\varepsilon_{\mathrm{si}}}/\left(1 + \sqrt{\varepsilon_{\mathrm{si}}}\right)$, $t_{23} = 2/\left(1 + \sqrt{\varepsilon_{\mathrm{si}}}\right)$, $r_{12} = -r_{23} = \left(1 - \sqrt{\varepsilon_{\mathrm{si}}}\right)/\left(1 + \sqrt{\varepsilon_{\mathrm{si}}}\right)$, and $k_{z,\mathrm{si}} = \sqrt{\varepsilon_{\mathrm{si}}}\omega/c$, where c is the speed of light in vacuum.

The difference between the full diffusion model and the average carrier density approximation is shown in Fig. S2, in which we have calculated the magnitude of the difference as a function of the surface recombination velocity for a range of bulk lifetimes, and for the same choices of wavelength of the photoexciting light and wafer thickness as used in Fig. S1. The frequency of the transmitted

radiation was chosen to be 65 GHz so that for the 675 µm thick wafer the transmission is resonantly enhanced by the Fabry-Perot condition as discussed in the accompanying article.

For the 100 µm thick wafer the difference in transmission between the full diffusion model and the average carrier density model is always very small, being a tiny fraction of a percentage. This is the case even when there would be a large variation in the carrier density through the thickness of the wafer. Take, for example, the carrier density profile in Fig. S1c for a surface recombination velocity of 1000 m/s, in which the carrier density varies by approximately 50 times through the thickness of the wafer. Even with this large variation in carrier density the difference in transmission between the two models is still extremely small.

For the 675 µm thick wafer there is a larger difference between the models. This is due to the resonant enhancement of the transmission as a result of the Fabry-Perot condition. Since, on resonance, the time-averaged magnitude of the electric field profile within the wafer will be non-uniform, all regions of the wafer are not sampled equivalently. In addition, since the Fabry-Perot resonance arises from the coherent addition of multiple reflections from the wafer surfaces, it will be more sensitive to variations in carrier density in these regions, exactly as is evident in the carrier distributions of Fig. S1. However, even so, the difference between the models is less than 1% for a very wide range of bulk lifetimes and surface recombination velocities, justifying our use of the simplified theory in the accompanying article.

As a final comment, the sensitivity of the transmission upon Fabry-Perot resonance to the carrier density near the surfaces is the reason why the transmission through the field-effect passivated wafers in the accompanying article cannot be accurately represented by either a uniform carrier density model, nor the full diffusion model described above. The passivation using Al_2O_3 induces a very thin (<100 nm) region of hole accumulation with densities up to 10^{25} m^{-3}, with a corresponding reduction in electron density (see [5]). This very large change in the carrier densities near the surface is the cause of the 10% reduction in the transmission on resonance in Fig. 4b of the accompanying article as compared to the unpassivated wafer in Fig. 4a. In order to accurately model such a system a far more complex model that treated electrons and holes separately, and that incorporated Auger recombination and the surface charge associated with the Al_2O_3, would be required.

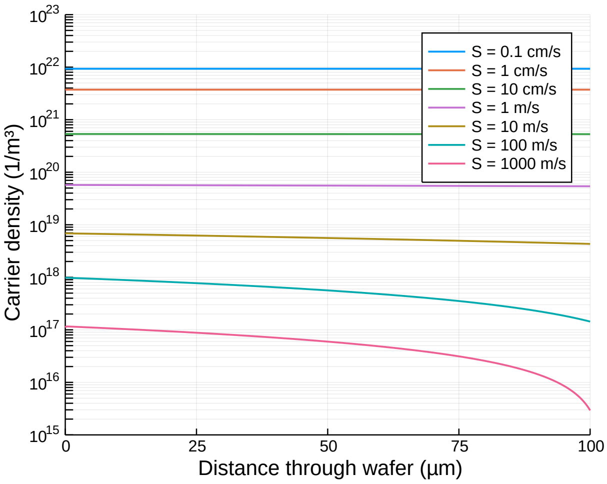

(a)

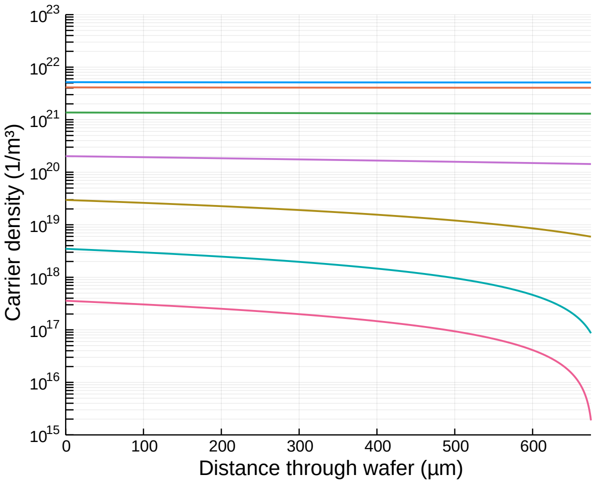

(b)

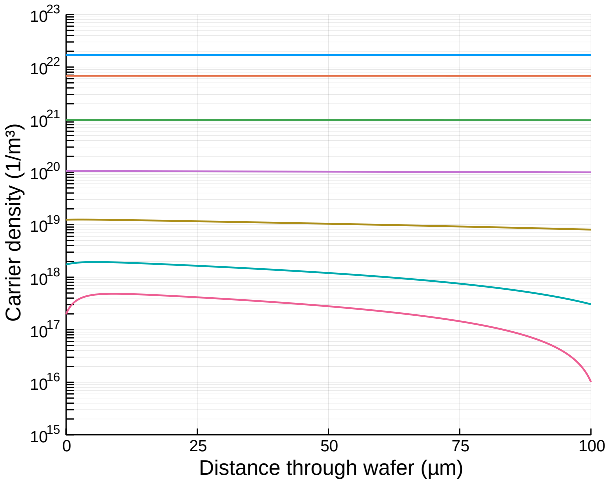

(c)

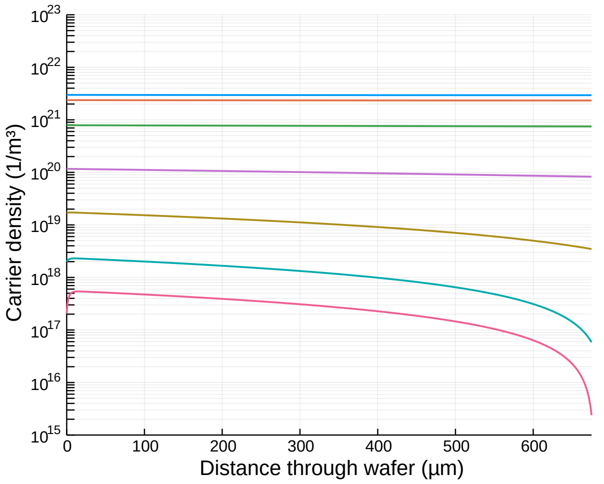

(d)

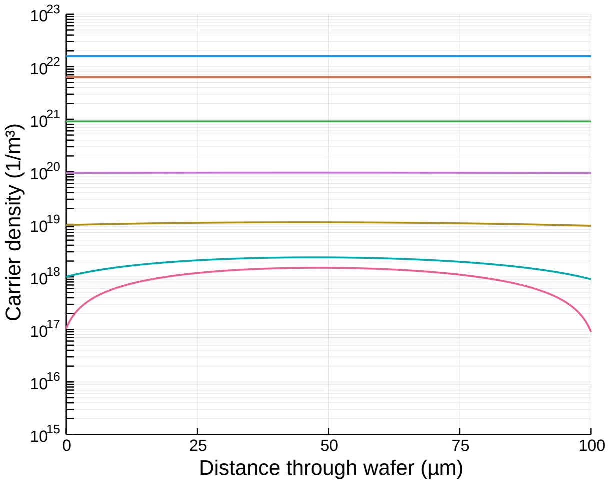

(e)

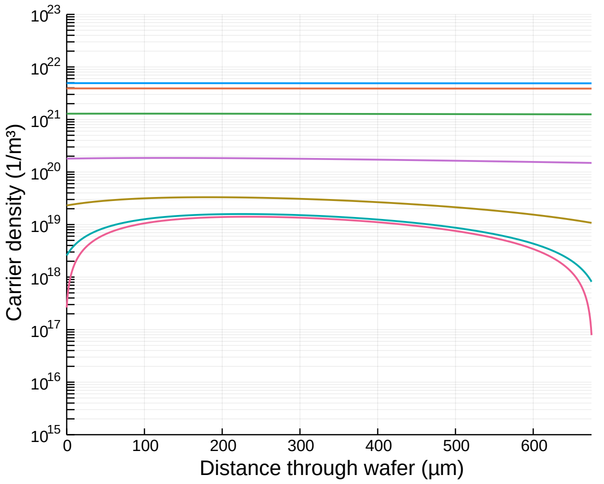

(f)

Figure S1: The charge carrier density profile as a function of the distance through the wafer for various surface recombination velocities. (a, c and e) have a wafer thickness of 100 μm, whilst (b, d, and e) have a wafer thickness of 675 μm. The wavelength of the photoexciting light is (a and b) 400 nm, (c and d) 625 nm, and (e and f) 1000 nm. The bulk lifetime is 10 ms, and the background carrier density is 10^{15} m^{-3}

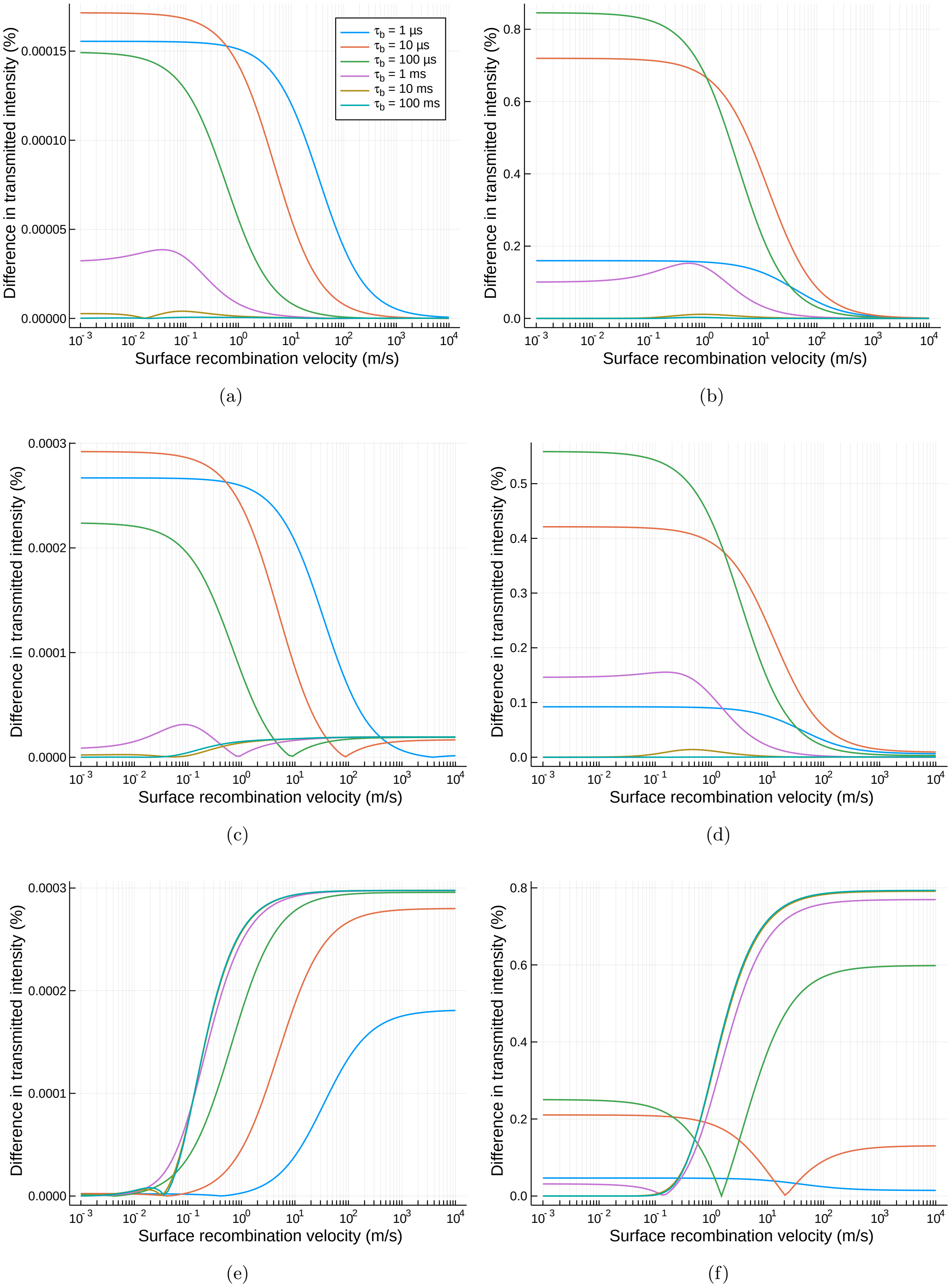

Figure S2: The difference in the transmission of 65 GHz radiation through a silicon wafer as a function of surface recombination velocity and for various bulk lifetimes between the full diffusion model and a model using only the average carrier density. The wafer thickness and wavelength of the photoexciting light is the same as in Fig. S1, as is the background carrier density.

4 Effective carrier lifetimes

In the accompanying article we state (in the text discussing recombination processes and referring to Eqn. 6) that in unpassivated wafers the effective lifetime of photoexcited carriers is dominated by recombination at the surface. In this section we calculate the effective carrier lifetime from spatially dependent carrier distributions obtained using the model described above, and show that surface recombination is indeed the dominant factor in high-quality wafers with high bulk lifetimes.

We start with Eqn. 2 from the accompanying article,

$$\Delta N = G\tau_{\text{eff}} \tag{24}$$

where ΔN is the average carrier density through the wafer, τ_{eff} is the average lifetime of a charge carrier, and $G = TI/(hfd)$ is the generation rate of the electron-hole pairs (this is simply the areal number of charge carriers generated throughout the wafer divided by its thickness), and assumes that the thickness of the wafer is greater than the penetration depth of the exciting light into the wafer, as in the accompanying article.

Thus, we can determine the effective carrier lifetime from a spatial carrier density profile by simply taking the average carrier density and dividing it by the (known) generation rate.

In Fig. S3 we show the effective carrier lifetime as a function of the surface recombination velocity for various bulk lifetimes for three different excitation wavelengths and two different wafer thicknesses, as in the previous section. The surface recombination velocity is the same for both interfaces.

For each of the plots we can identify three regions. 1) At low surface recombination velocities the effective lifetime approaches the bulk lifetime, as expected. 2) For intermediate surface recombination velocities there is a rapid reduction in the effective lifetime as recombination at the surfaces begins to dominate. 3) At very high surface recombination velocities the effective lifetime "flattens out" once more. This occurs when charge carriers excited in the bulk of the wafer recombine sufficiently quickly that they never encounter the surfaces. This is particularly noticeable in Fig. S3f where much of the carrier generation occurs away from the front surface due to the long penetration depth (approximately 160 µm) of 1000 nm wavelength radiation. (Note that the reduction in the low surface recombination velocity asymptotic limits in Fig. S3e is erroneous and results from the approximation that the generation rate is independent of the wafer thickness, as described above. Since the penetration depth of the photoexciting light is longer than the 100 µm thickness of the wafer, the effective lifetime is underestimated).

Whilst these plots show that, in general, the effective carrier lifetime is strongly dependent upon the wafer thickness and excitation wavelength, as well as the bulk and surface lifetimes, for high bulk lifetime wafers, as studied in the accompanying article, surface recombination dominates the effective lifetime. They also show that unpassivated wafers, which will have surface recombination velocities of the order of 10^3-10^4 m/s, will exhibit effective lifetimes of the order of sub-µs to 10s of µs depending upon the wafer thickness and excitation wavelength.

5 References

1. Rosling, M., Bleichner, H., Jonsson, P., & Nordlander, E., The ambipolar diffusion coefficient in silicon: Dependence on excess-carrier concentration and temperature, *Journal of Applied Physics*, **76**, 2855, DOI:10.1063/1.358504 (1994).

2. Kannegulla, A., Shams, M. I. B., Liu, L. & Cheng, L.-J. Photo-induced spatial modulation of thz waves: opportunities and limitations. *Opt. express*,**23**, 32098–15, DOI: 10.1364/OE.23.032098 (2015).

3. Klaassen, D. A unified mobility model for device simulation—i. model equations and concentration dependence. *Solid-State Electron.***35**, 953–959, DOI: https://doi.org/10.1016/0038-1101(92)90325-7 (1992)

4. Schinke, C. *et al.* Uncertainty analysis for the coefficient of band-to-band absorption of crystalline silicon. *AIP Advances* **5**, 67168, DOI:10.1063/1.4923379 (2015).

5. Dingemans, G. & Kessels, W. M. M. Status and prospects of Al_2O_3-based surface passivation schemes for silicon solarcells. *J. Vac. Sci. & Technol. A: Vacuum, Surfaces, Films*, **30**, 040802–28,

DOI: 10.1116/1.4728205 (2012).
6. Cuevas, A. & Macdonald, D. Measuring and interpreting the lifetime of silicon wafers. *Sol. Energy*, **76**, 255–262, DOI:10.1016/j.solener.2003.07.033 (2004).

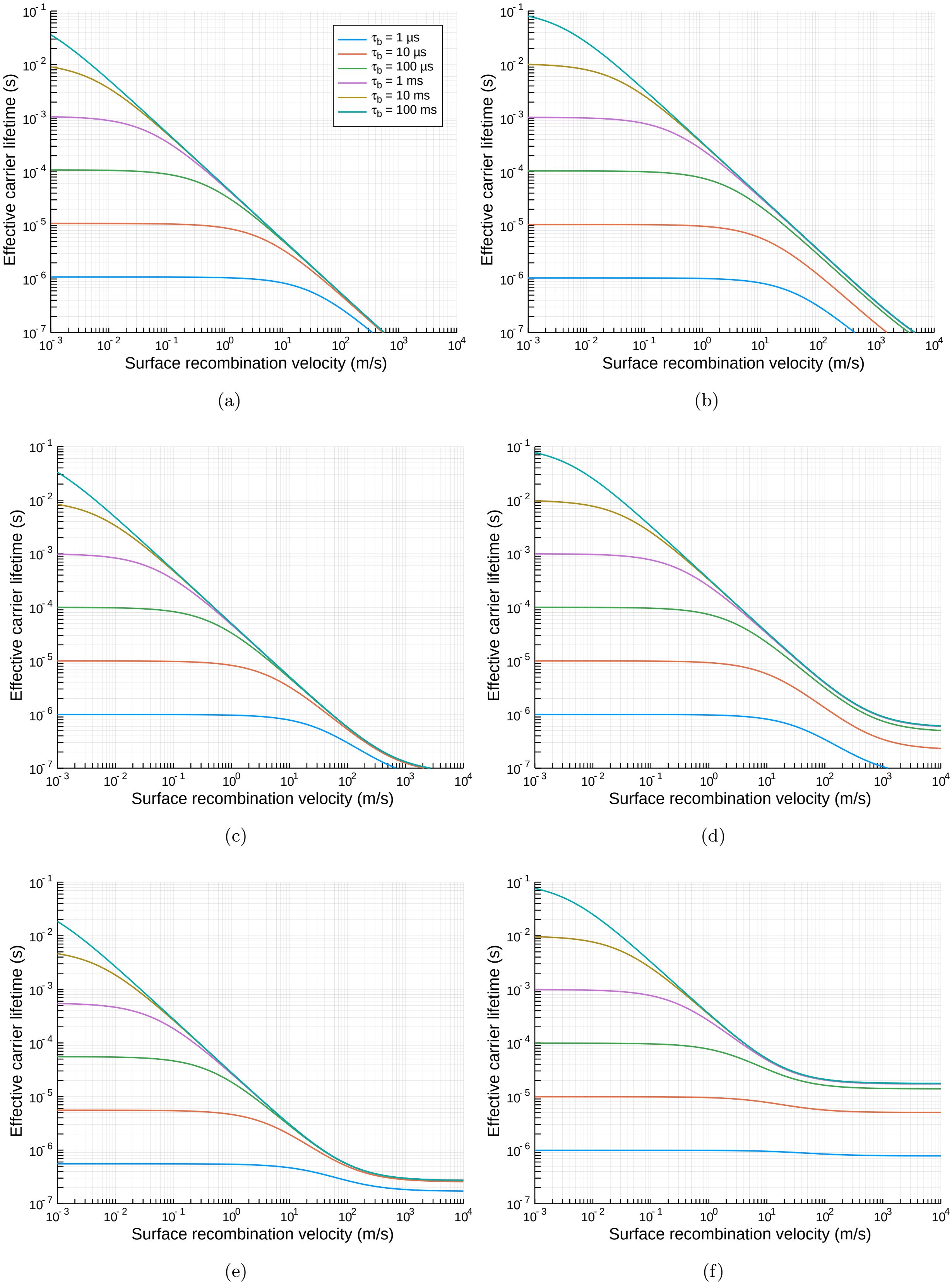

Figure S3: The effective carrier lifetime as a function of surface recombination velocity and for various bulk lifetimes as calculated from the average carrier density. The wafer thickness and wavelength of the photoexciting light is the same as in Fig. S1, as is the background carrier density.
